# Musculoskeletal Model Development of the Elbow Joint with an Experimental Evaluation

**DOI:** 10.3390/bioengineering5020031

**Published:** 2018-04-20

**Authors:** Munsur Rahman, Mohsen Sharifi Renani, Akin Cil, Antonis P. Stylianou

**Affiliations:** 1 Department of Civil and Mechanical Engineering, University of Missouri-Kansas City, 5110 Rockhill Road, Kansas City, MO 64110, USA; Mohsen.SharifiRenani@du.edu (M.S.R.); Akin.Cil@tmcmed.org (A.C.); stylianoua@umkc.edu (A.P.S.); 2 Department of Orthopaedic Surgery, University of Missouri-Kansas City, 2411 Holmes Street, Kansas City, MO 64108, USA; 3 Department of Orthopaedics, Truman Medical Centers, 2301 Holmes Street, Kansas City, MO 64108, USA

**Keywords:** musculoskeletal model, elbow joint, cartilage, ligaments, contact mechanics, kinematics, upper extremity

## Abstract

A dynamic musculoskeletal model of the elbow joint in which muscle, ligament, and articular surface contact forces are predicted concurrently would be an ideal tool for patient-specific preoperative planning, computer-aided surgery, and rehabilitation. Existing musculoskeletal elbow joint models have limited clinical applicability because of idealizing the elbow as a mechanical hinge joint or ignoring important soft tissue (e.g., cartilage) contributions. The purpose of this study was to develop a subject-specific anatomically correct musculoskeletal elbow joint model and evaluate it based on experimental kinematics and muscle electromyography measurements. The model included three-dimensional bone geometries, a joint constrained by multiple ligament bundles, deformable contacts, and the natural oblique wrapping of ligaments. The musculoskeletal model predicted the bone kinematics reasonably accurately in three different velocity conditions. The model predicted timing and number of muscle excitations, and the normalized muscle forces were also in agreement with the experiment. The model was able to predict important in vivo parameters that are not possible to measure experimentally, such as muscle and ligament forces, and cartilage contact pressure. In addition, the developed musculoskeletal model was computationally efficient for body-level dynamic simulation. The maximum computation time was less than 30 min for our 35 s simulation. As a predictive clinical tool, the potential medical applications for this model and modeling approach are significant.

## 1. Introduction

As an important joint of the upper extremity, the elbow joint serves as a fulcrum of the forearm lever that greatly enhances the spatial positioning of the hand. Because of the centrality of the elbow joint to the upper extremity system, loss of or diminished function of the elbow joint results in significant deficits in upper extremity function, and can jeopardize individual independence [1]. Therefore, biomechanical analysis of the elbow joint is extremely important in understanding elbow injuries, better execution of trauma management, and prosthetic design.

Detailed knowledge of the in vivo loading of elbow structures is essential in understanding the biomechanical causes associated with both chronic (degenerative joint disease) and acute injuries, and for improving the design and implementation of therapeutic interventions. Since the direct measurement of the in vivo joint loads is not technologically feasible, computational models need be implemented for predictions. A dynamic computational elbow joint model capable of concurrent prediction of muscle and ligament forces along with the cartilage contact mechanics would be an ideal tool in clinical practice. Once the model is validated, multiple disease states can be tested to improve our clinical understanding of the elbow pathology, test the current elbow implants, and design a better implant for partial or total elbow replacements. In addition, the model could provide subject-specific intervention strategies aimed at modifying upper extremity movement for targeted outcomes, such as reducing articular cartilage stress. Computational models can also mitigate the need for large sample sizes in clinical trials and can work at a fraction of the cost of cadaveric models.

Computational models of the elbow have been employed to study the joint biomechanical behaviour and analyse musculoskeletal movement [2,3,4,5]. However, these models have limited clinical applicability by assuming a fixed joint axis of rotation (e.g., hinge joint) rather than a true anatomical joint constrained by ligament forces and cartilage contacts. In some circumstances, such simplifications would be helpful; however, the human elbow joint has significant laxity that should not be ignored [6,7]. In a recent study, Fisk and Wayne [8] developed an anatomical model of the elbow joint where the joint behaviour was dictated by ligament constraint and articular contact. However, there are still assumptions that have been made in this advanced model that cannot fully replicate the normal elbow physiology/clinical scenarios. The significant limitations of this model are that the model did not include articular cartilage, the ligaments were modelled as linear springs, the muscles were represented as constant-magnitude force vectors, and the wrapping of ligaments and muscles around the bony structures was ignored. The need for dynamic anatomically correct computational elbow joint models that link muscle forces, motion, and joint contact characteristics has long been recognized. To our knowledge, such an advanced musculoskeletal elbow joint model does not exist in the literature.

The objective of this study was to develop an anatomically correct musculoskeletal elbow joint model and evaluate it based on experimental kinematics and muscle electromyography (EMG) measurement. The joint was constrained by multiple nonlinear ligament bundles and three-dimensional deformable contacts. Moreover, the model included the natural oblique wrapping of muscle and ligaments around the joints. The developed elbow model is a step toward developing a complete musculoskeletal model of the upper extremity.

## 2. Materials and Methods 

### 2.1. Experimental Measures

**Figure 1:**
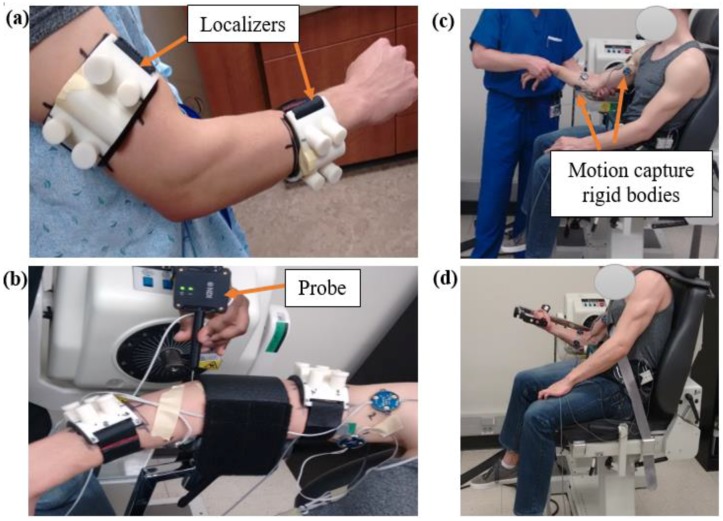
Experimental measurement: (**a**) Two plastic localizers attached to subject upper and lower arm; (**b**) Initial position collection using Optotrak probe; (**c**) The experimenter manipulating the arm for laxity test; (**d**) The subject performing the experimental trial.

One healthy volunteer (24 years old, male) with no history of upper extremity problems was recruited for the project after providing written informed consent approved by the institutional human subject review board. Two localizers made of ABS plastic were attached to the subject’s upper arm and the forearm; the localizers included two orthogonal tubes (Figure 1a). The tubes were filled with fluid (mustard) that appeared in the magnetic resonance images (MRIs). The localizers, along with the tubes, were used to register the coordinate system of the bone, cartilage, ligament, and muscle geometries later in the modelling process. High-resolution MRI (Siemens 1.5T machine, TR: 14.8, TE: 6.18, slice thickness 0.5 mm, imaging frequency 63.63 Hz, and group lengths 178) was acquired on the subject’s dominant upper extremity. The localizers were not removed as the subject travelled to the UMKC Human Motion Lab. Two Optotrack motion capture rigid bodies (Northern Digital Inc, Waterloo, Ontario, Canada) were attached to the subject’s upper arm and two rigid bodies were attached to the subject’s lower arm to collect the motion data. Each rigid body contains three infrared markers to capture 6 degrees of freedom of body segment (3 translations and 3 rotations). 

The subject was positioned on a Biodex Multi-Joint Dynamometer system (Biodex Medical Systems, Shirley, NY, USA) where the dominant arm was kept in a pronated rest position (Figure 1b). The initial position and orientation of the arm were determined by recording the coordinates of multiple points on the localizers, on the bony landmarks of the arm, and along the arm surface by using an Optotrak probe tool. Following collection of the initial position, the localizers were removed, and an Orthopaedic surgeon performed a standard laxity test for the elbow (Figure 1c). This test involved moving the elbow joint through its full range of motion (flexion–extension and varus–valgus) by applying minimal force to the joint while motion data from the body segments were collected. This established the kinematic range of motion data from which the ligament zero-load lengths (the lengths at which the ligament first become taut) were extracted. The dynamic testing of the elbow was performed in the dynamometer (Figure 1d). Along with the Optotrack markers, the subject was also outfitted with two surface EMG sensors (Delsys Myomonitor IV wireless EMG system, Delsys Inc., Natick, MA, USA) on the middle of the muscle as suggested for bicep and tricep muscles to record muscle activity [9,10]. The subject was asked to perform three seated elbow flexion/extension trials in three different protocols: (1) isokinetic at 10 deg/s, (2) isokinetic at 60 deg/s, and (3) at self-selected angular velocity (as fast as possible by the subject). For each trial, arm segment motions (at 100 Hz) and EMG activity (at 1400 Hz) were recorded.

### 2.2. Computational Model

**Figure 2:**
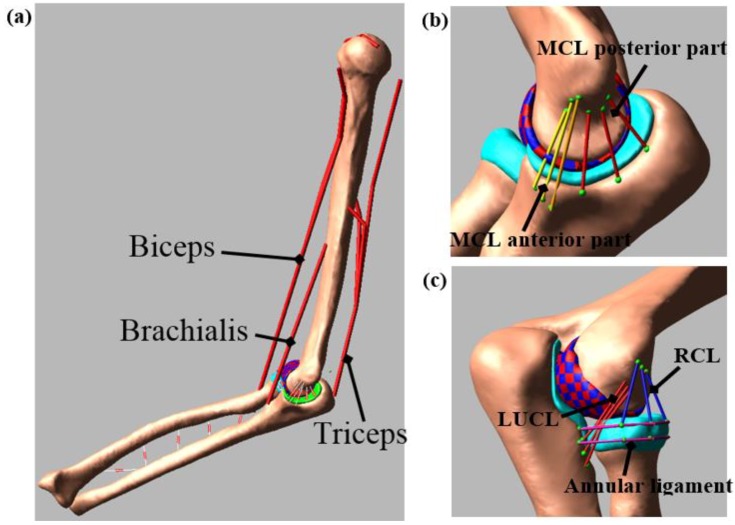
Multibody Model: (**a**) A subject-specific model representing bones and major muscles crossing the elbow joint, (**b**) medial collateral ligament (MCL) complex, and (**c**) lateral collateral ligament complex in the model.

Three-dimensional bone and cartilage geometries were generated from the MRI using 3D Slicer (www.slicer.org). The geometries were imported to MeshLab for postprocessing that included removing the spikes, reducing the noise, and smoothing the surface [11]. The multibody model was created in ADAMS (MSC Software Corporation, Santa Ana, CA, USA) by aligning these geometries using the initial position and orientation obtained during the experimental study (Figure 2a). The bone and the cartilage densities were set at 1600 kg/m^3^ [12] and 1000 kg/m^3^ [13], respectively.

The ligaments and the interosseous membranes were modelled as a different number of bundles based on their structure and function. The model included three bundles for the medial collateral ligament (MCL) anterior part [14], three bundles for the MCL posterior part, three bundles for the lateral ulnar collateral ligament (LUCL) [15], three bundles for the radial collateral ligament (RCL) [8], and two bundles for the annular ligament (Figure 2b,c). The ligaments were attached to the bone, according to the attachment sites identified in the MRI and in published studies [1,8,16,17]. Each ligament bundle was modelled as tension-only nonlinear springs using a piecewise function that includes the “toe” region [18,19]. The toe region simulates the crimping effect of the ligament which represents the parabolic transition between the zero strain and the linear region of the ligament. 

The force-length relationship for each ligament bundle is described by Equations (1) and (2):

(1) $$f=\{\begin{array}{cc} \frac{1}{4}k\varepsilon^{2}/\varepsilon_{l} & \;0\leq \varepsilon \leq 2\varepsilon_{l}\\ k\left(\varepsilon -\varepsilon_{l}\right) & \;\varepsilon >2\varepsilon_{l}\\ 0 & \varepsilon <0 \end{array}$$

(2) $$\varepsilon =\left(\frac{l-l_{0}}{l_{0}}\right)$$

where *k* is the stiffness parameter, $\varepsilon_{l}$ is a spring parameter assumed to be 0.03 [20], *l* is the length of the each bundle, and *l*_0_ is the zero-load length [19,21]. The ligament stiffness parameter was obtained from the literature [8,22], and the zero-load length was calculated based on the laxity test. The zero-load length for each ligament bundle was determined by the maximum distance measured between ligament insertion and origin sites throughout the motion range during the laxity test and then multiplied by a correction factor (80% of max length) [23]. This correction factor reduced the error unintentionally introduced by the experimenter when applying a small amount of ligament force. The ligaments were wrapped around the bone to represent their anatomical physiology, and to prevent the penetration of the ligament into the bone [24]. 

A custom macro was written in ADAMS to automatically divide the humerus cartilage into discrete hexahedral elements. The macro performance was successfully tested in many of our previous lab studies [15,24,25,26,27,28,29]. Each cartilage element had an approximate 3 × 3 mm cross-sectional area. The macro also defined a deformable contact constraint with no friction using an ADAMS compliant contact model (Equation (3)) between each humerus cartilage element with the radius and ulna cartilage geometry:

(3) $$F_{c}=k_{c}\delta^{n}+B_{c}\left(\delta \right)\dot{\delta }$$

where *k_c_* is the contact stiffness, *δ* is the interpenetration of the geometries, and *B_c_(δ)* is a damping coefficient. Optimization and design of the experiment approach were used to determine the contact parameters and the size of discretized cartilage elements from a cadaver study (Table 1). The optimization was done in such a way that the maximum contact pressure and contact area errors were minimized between a multibody model and an identically loaded finite element model [27,30].

**Figure 3:**
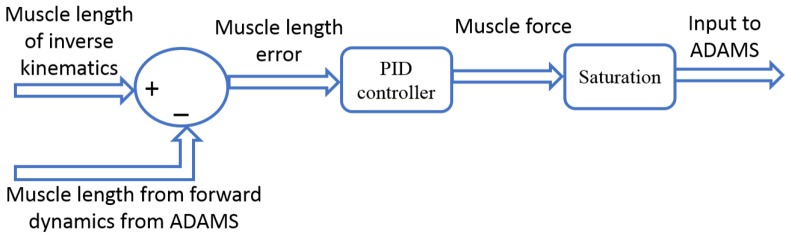
Feedback control scheme for calculating muscle force.

Since the model evaluation did not require the complete set of muscle, the model included three major muscles that cross the elbow joint: triceps (long, lateral, and medial), biceps (long, short), and brachialis. Muscle modelling mechanical parameters, insertions, origins, and via-points are based on published literature [4]. The musculoskeletal model simulation was executed in two phases. First, during the inverse kinematics, the experimental motion data were used to move the model as constrained by the joint contacts and ligaments. The shortening/lengthening pattern of each muscle element was recorded during this step. Next, the kinematic constraints were removed, and the muscles served as actuators during forward dynamics. The muscle forces were calculated via proportional–integral–derivative (PID) controllers implemented in Simulink (The MathWorks, Inc., Natick, MA, USA). During the forward dynamics simulation, ADAMS and Simulink were linked in co-simulation. In the process, ADAMS sent the current muscle lengths to Simulink and Simulink then sent the muscle forces to ADAMS for the next step of calculation (Figure 3) [25]. The error signal between the current forward dynamics muscle length and the muscle length measured during the inverse kinematics simulation was minimized by the PID controllers. The output of the PID controller was the muscle forces for the forward dynamic simulation to track the inverse kinematics muscle length. 

The muscle force was limited in a way that it can only pull, not push. In addition, the PID parameters for each individual muscle were scaled based on the following equation:

(4) $$P^{i}=\frac{{\mathrm{PCSA}}^{i}}{\mathrm{Reference}\;\mathrm{PCSA}}\times \mathrm{Global}\;\mathrm{Pi}=1,2\ldots .\#\mathrm{of}\;\mathrm{muscles}$$

where P^i^ is the proportional gain for muscle i. The PCSA^i^ term is the physiological cross-sectional area of each muscle and originates from the work by Holzbaur et al. [4]. The reference PCSA = 487 mm^2^ was calculated as the average of all muscles. Similar equations were also applied for the integral and derivative gains. The global PID values for the muscle controller were P = 50, I = 5, and D = 0.0005 [25]. Muscles with a PCSA less than the reference PCSA will have smaller PID gains while larger muscles will have larger PID gains. Furthermore, the force generated by an individual muscle was limited by its maximum force generating capacity.

Local coordinate systems for each bone segment were created to measure the ulna and radius motions relative to the humerus [27]. The translations were represented as medial–lateral (M–L), anterior–posterior (A–P), and superior–inferior (S–I) directions and the rotations were represented as flexion–extension (F–E), varus–valgus (VR–VL), and internal–external (I–E) rotation. The model was evaluated by comparing the model-predicted kinematics (forward dynamics) to the experimental measurements (inverse kinematics). The predicted muscle activation patterns were also compared to the experimental EMG measurements. The experimental EMG signals were demeaned, rectified, and then low-pass filtered to eliminate measurement noise using a second-order Butterworth low-pass filter with a cut-off frequency of 6 Hz. Then, the filtered EMG signal was normalized to the maximum value of the specific trial for each muscle. We calculated the root mean square (RMS) error and correlation coefficient to compare the model-predicted kinematics with experimental results. After model evaluation, ligament loads, joint contact locations, contact area, and pressures were predicted from the musculoskeletal model simulation.

## 3. Results

### 3.1. Model Evaluation

**Figure 4:**
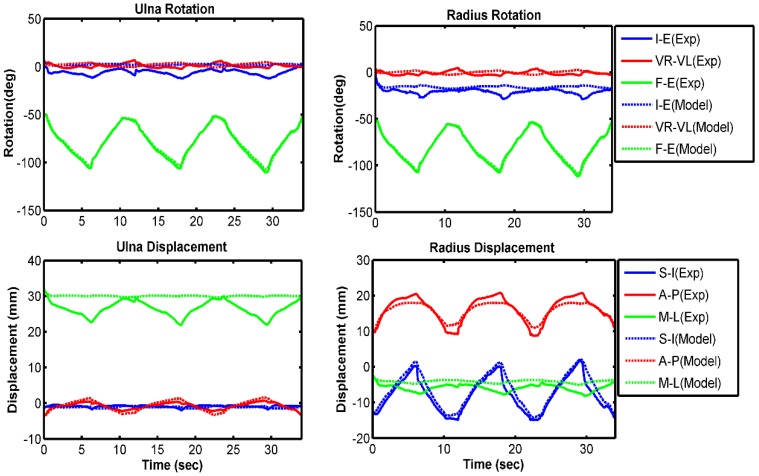
Comparison of bone kinematics between experiment and model prediction for 10 deg/s trial. Increasing trend of the graph indicates more internal rotation for I–E, more valgus rotation for VR–VL, and more extension for F–E. Similarly, it indicates more superior translation for S–I, more anterior translation for A–P, and more medial translation for M–L. Decreasing trend of the graph indicates the opposite.

**Figure 5:**
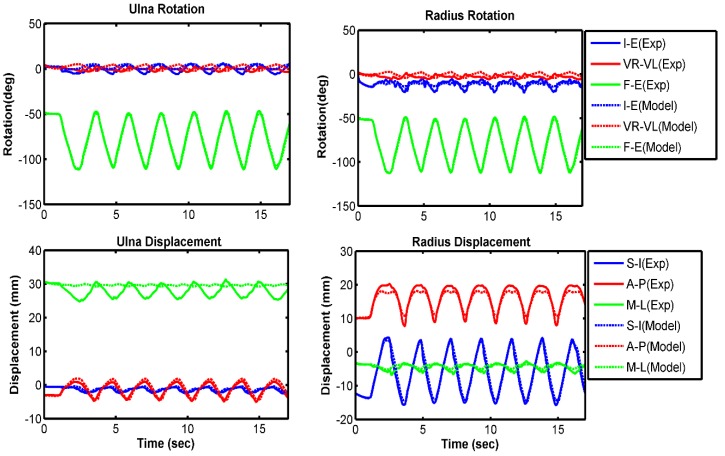
Comparison of bone kinematics between experiment and model prediction for 60 deg/s trial. Increasing trend of the graph indicates more internal rotation for I–E, more valgus rotation for VR–VL, and more extension for F–E. Similarly, it indicates more superior translation for S–I, more anterior translation for A–P, and more medial translation for M–L. Decreasing trend of the graph indicates the opposite.

**Figure 6:**
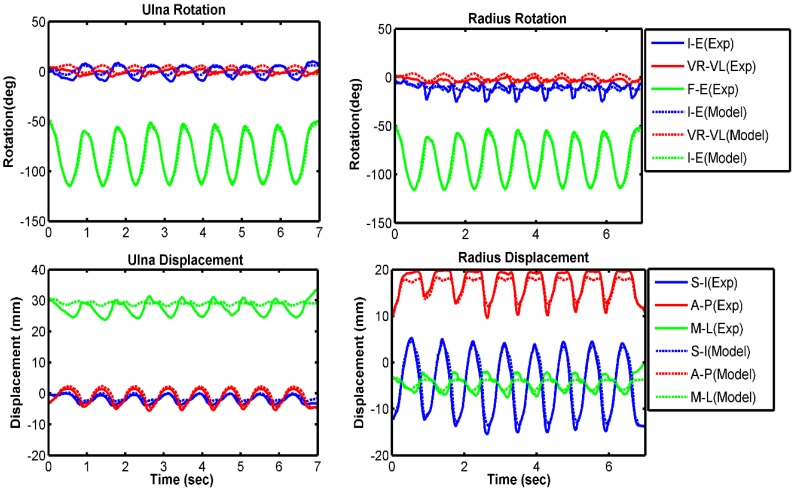
Relative bone kinematics for experiment and model prediction for the free velocity trial. Increasing trend of the graph indicates more internal rotation for I–E, more valgus rotation for VR–VL, and more extension for F–E. Similarly, it indicates more superior translation for S–I, more anterior translation for A–P, and more medial translation for M–L. Decreasing trend of the graph indicates the opposite.

Kinematic comparisons between experimental results and model predictions are presented in each anatomical direction for the ulna and radius relative to the humerus (Figure 4, Figure 5 and Figure 6). Overall, the model compared well with the experiment for all three velocity conditions of 10 deg/s, 60 deg/s, and free speed. The forearm bones rotated 3° to 7° more internally in the model prediction than in the experiment (Table 2). The model also predicted 2°–3° greater valgus rotation and 2–4 mm lower lateral translation compared with the experimental measurement. All other model-predicted kinematics followed the experiment very well. Overall, we observed higher RMS errors at lower speeds of forearm movement. On average, 8 out of 12 kinematics had a good correlation (greater than 80%) between model and experiment. 

**Figure 7:**
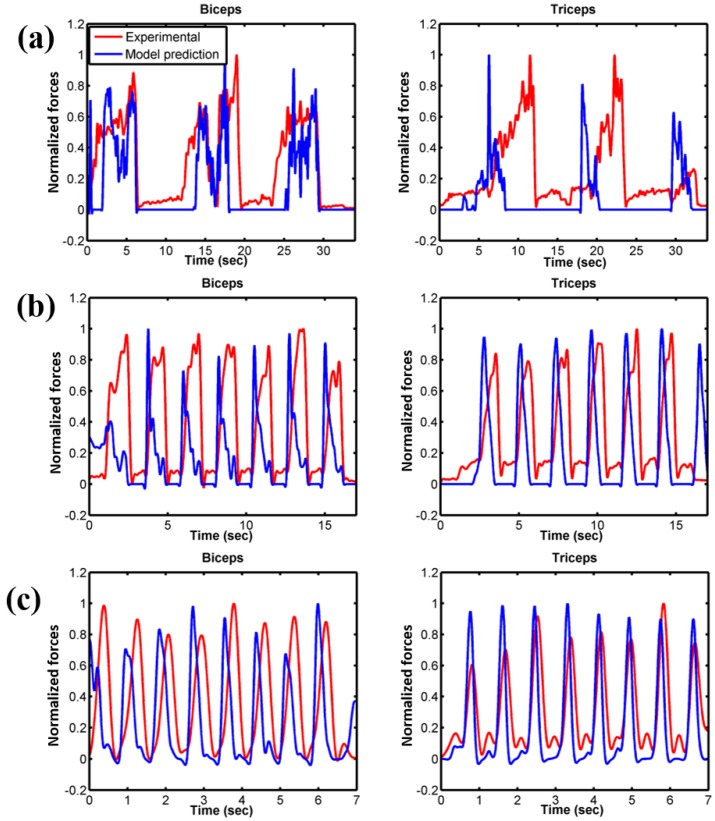
Normalized experimental electromyography (EMG) and normalized muscle forces from model prediction. (**a**) 10 deg/s; (**b**) 60 deg/s; (**c**) free velocity. Muscle forces are normalized to the maximum force produced by each muscle for the specific trial.

For each velocity condition, the timings when the muscle started to activate in each loop were similar both in model prediction and experimental muscle excitation measured from EMG (Figure 7). The total numbers of muscle activations were also consistent between the model and experiment. In addition, most of the normalized peak forces were in agreement between model and experiment. However, in our model prediction, the maximum muscle contraction occurred faster than the experimental measurement; therefore, the muscle relaxation also started earlier. Although more pronounced, the model was able to reproduce the trend very well. 

### 3.2. Force Prediction

**Figure 8:**
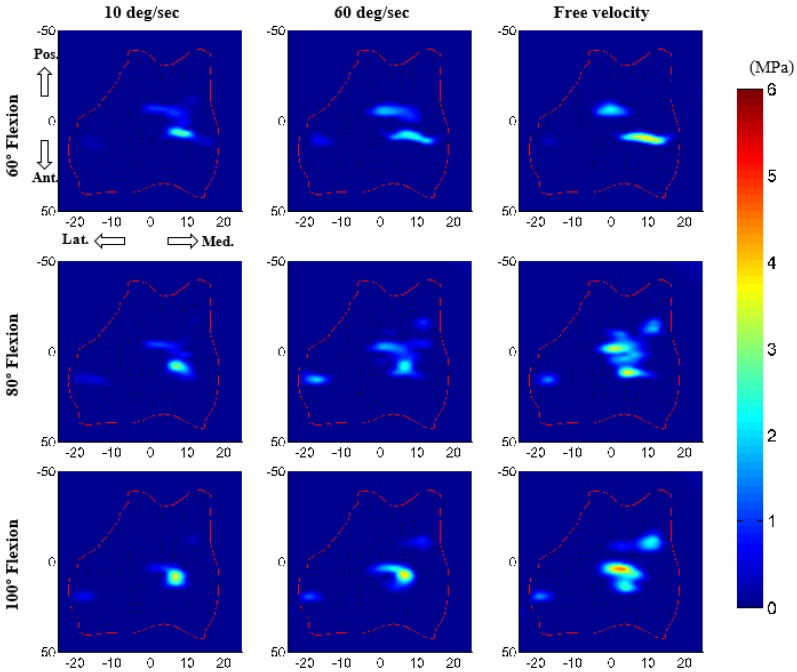
Contact pressure distribution on humeral cartilage for muscle-driven forward dynamic simulation. *x-* and *y*-axes units are in mm and the intensity of colour represents the intensity of contact pressure. The red boundary represents the unfolded 2D projection of the humerus cartilage.

The core advantage of the computation model is its ability to predict important parameters that are very hard or impossible to measure experimentally, such as ligament force and cartilage contact pressure. In this study, the model predicted noticeably increased peak ligament loads with increasing forearm velocity (Table 3). Contact pressure distributions on the humeral cartilage were also considerably different for various velocity conditions (Figure 8). Both contact area and contact pressure were noticeably increased with increasing velocity. Peak contact pressure (on medial cartilage) was 3.7 MPa for 10 deg/s, 4.2 MPa for 60 deg/s, and 5.5 MPa for free velocity. As expected, contact areas were much higher for the ulnohumeral joint (medial contact) than for the radiohumeral joint.

Computation time for our longest 35 s simulation (10 deg/s condition) was less than 30 min (0.01 s step size, default ADAMS solver, desktop PC (Intel®Xenon®CPUE5-16070@3.00GHz with 32 GB of RAM). Simulation times for the other two cases were even smaller.

## 4. Discussion

The principal goal of this study was to develop an anatomically correct subject-specific musculoskeletal model of the elbow joint. The model was evaluated by comparing the bone kinematics from the model simulation (muscle-driven forward dynamics) to the experimental motion-captured kinematics. As an additional evaluation, the normalized muscle forces were also compared with the experimental normalized EMG data. The model included three-dimensional bone and cartilage geometries. The elbow joint was constrained by nonlinear ligament bundles and three-dimensional deformable contacts, instead of an idealized mechanical joint. The model also included the natural oblique wrapping of muscle and ligaments. Articular contact force and contact area predictions were achieved by discretizing the humerus cartilage into multiple hexahedral elements. An optimization and the design of the experiment approach were used to determine the contact parameters and the size of discretized cartilage elements. The developed musculoskeletal model is capable of concurrently predicting muscle and ligament forces, along with cartilage contact mechanics during dynamic activities. Such concurrent prediction from an anatomical model has the potential to be a powerful predictive tool in Orthopaedics. The model is also very computationally efficient for body-level dynamic simulation. Such a simulation in finite element analysis would take an enormous amount of time to solve. 

Previously, computational musculoskeletal models have been employed to predict the muscle activation and upper limb strength by constraining the elbow joint as a single one-degree-of-freedom mechanical joint [31,32,33]. However, this assumption removes the influence of contact forces on muscle forces and muscle contributions to motion beyond the sagittal plane. Because of the interdependency between the articular contact and the muscle forces, those forces need to be computed concurrently. Furthermore, three-dimensional measurements of simulated active elbow motion by an electromagnetic tracking device revealed the amount of potential varus-valgus laxity that occurred during elbow flexion to be about 3 to 4 degrees [7]. Ignoring this laxity by placing a mechanical joint can affect the muscle force predictions. In addition, the omission of this normal laxity into the implant design is the reason behind the failure of fully constrained elbow replacement implants, because it increases the stress transfer to the implant-cement-bone interfaces and results in aseptic loosening [34]. Therefore, an accurate elbow model should reflect the intrinsic laxity of the elbow, especially for clinical applications. In this study, the elbow joint was constrained by the ligaments and the ulnohumeral, radiohumeral, and radioulnar contact forces allowing 18 degrees of freedom.

The model-predicted kinematics compared well with the experimental measurements. However, some variations were observed in kinematic comparison, especially in the varus–valgus directions (Table 2). We did not include the joint capsule in our model which may be one of the major contributing factors to this error. Morrey and An [35] reported that the anterior and posterior capsule provided 32% varus and 33% valgus elbow stability, respectively. The mechanical properties of the elbow joint capsule were not available in the literature, which limited its inclusion in our model. In addition, during a large range of flexion–extension, the Optotrak markers could slightly move from their initial position due to relative muscle movement from bone and may introduce some measurement artefacts. 

The activation patterns of each of the contributing major muscles were correctly identified for all velocity conditions and favourably compared with experimental EMG measurements (Figure 7). The small deviation of the muscle activation pattern may be due in part to the experimental measurement of EMG voltage. We were able to measure two surface EMGs from two muscle groups (triceps and biceps) of the upper arm. Isolating the specific muscle group during the experiment was extremely difficult; this might induce some cross-signal errors in the EMG measurement. In addition, only three muscles were powering the elbow flexion-extension in the model versus the total 24 muscles cross the elbow joint [1,36], which may influence the muscle to activate faster than in the real subject experiment and may cause phase difference. To our understanding, incorporating more muscles in the model could improve the muscle force prediction. 

Our predicted joint contact pressure was increased by increasing the arm flexion–extension velocity. This appears reasonable since at higher velocity the muscle forces are expected to increase, which would lead to higher compression at the joint and increased contact pressure. This is also consistent with the increasing ligament load at a higher velocity of the arm. Although the experimental measurement of joint contact pressure is not feasible from a live subject, our external prediction of joint kinematics and muscle activation replicate the experiment, which gives some confidence of our internal joint contact pressure prediction. Furthermore, our predicted ulnohumeral contact and noncontact areas were consistent with the contact patterns reported by Eckstein et al. [37] and the maximum contact pressures were close in range to the values (0–5 MPa) reported by Brand [38]. Overestimation of the pressure may be explained by the choice of the ligament zero-load length. The correction factor used in our study was constant, which may not be ideal for all ligament bundles [23] and may introduce some ligament tightness. 

A potential limitation of the present model was that it was developed based on a single-subject experiment and is therefore characteristic of a single elbow. A larger sample size would allow more generalized conclusions to be made for clinical applications. However, the modelling techniques, efficiency, and accuracy were successfully tested by the research team for five cadaver studies [14,24] and three studies of live subject musculoskeletal modelling of a lower extremity [25,26,28]. Furthermore, the model included only three major muscles that crossed the elbow joint. Other muscles cross either the shoulder or wrist joint along with the elbow joint. Therefore, a complete musculoskeletal model of the upper extremity containing shoulder, elbow, and wrist joints will allow a complete testing of hypotheses that describe the forces responsible for elbow joint loading. In addition, the muscle forces are computed using a feedback controller in this study. One problem with using a feedback controller is that it cannot predict muscle forces without changing the muscle length. Hybrid control methods where some EMG signals are used as input to the model may solve this issue. 

The advantage of the modelling approach and its applicability as a predicting tool greatly outweigh its limitation. To date, no other computational model has had the capability to predict the elbow joint kinematics, muscle and ligament forces, and cartilage contact pressure concurrently. Instead, past models have assumed a particular joint degree of freedom and ignored the effect of ligaments, cartilage, or physiological properties of the joint. This study developed a subject-specific musculoskeletal elbow joint model in which the joint was constrained by multiple ligament bundles, three-dimensional deformable contacts, and included the natural oblique wrapping of ligaments. The model has demonstrated a powerful ability to predict important parameters that are extremely difficult or even impossible to measure experimentally, such as muscle and ligament forces, and cartilage contact pressure. This model and modelling approach are a step toward developing a complete musculoskeletal model of the upper extremity. This method is not intended to replace existing models or modelling approaches, but rather to provide an additional supporting tool to understand the elbow joint functions and pathologies. Further refinements, such as more detailed subject-specific measurements and improvement of the musculature, can enhance its accuracy and clinical applicability. The potential medical application of this model and modelling approach is significant and is anticipated as a clinical tool for the development of patient-specific preoperative planning, computer-aided surgery, and computer-aided rehabilitation.

## Figures and Tables

**Table 1:** Optimized contact parameter information.

Parameters	Values
Contact type	Impact (deformable)
Contact element size	3 mm × 3 mm
Friction	No
Stiffness (*k_c_*)	40 N/mm
Damping coefficient (*B_c_(δ)*)	5 Ns/mm
Exponent (*n*)	3.05
Interpenetration of the geometries (*δ*)	0.1 mm

**Table 2:** RMS error (deg, mm) and correlation coefficients for ulna and radius kinematics (good correlations are in bold).

Kinematics Description	10 deg/s		60 deg/s		Free Velocity	
	RMS Error	Correlation Coefficient	RMS Error	Correlation Coefficient	RMS Error	Correlation Coefficient
Ulna I–E rotation	7.4	0.48	2.9	**0.86**	4.0	**0.92**
Ulna VR–VL rotation	2.8	−0.63	2.9	−0.13	2.7	0.57
Ulna F–E rotation	1.3	**0.99**	1.2	**0.99**	2.7	**0.99**
Radius I–E rotation	5.5	0.44	2.3	0.55	3.6	0.08
Radius VR–VL rotation	2.6	−0.60	2.8	−0.15	2.6	0.61
Radius F–E rotation	1.1	**0.99**	1.1	**0.99**	2.7	**0.99**
Ulna S–I displacement	0.4	0.07	0.2	**0.90**	0.4	**0.97**
Ulna A–P displacement	0.4	**0.98**	0.4	**0.99**	0.6	**0.98**
Ulna M–L displacement	3.4	−0.45	2.0	−0.62	2.3	−0.14
Radius S–I displacement	1.3	**0.99**	0.6	**0.99**	1.3	**0.99**
Radius A–P displacement	1.0	**0.98**	0.8	**0.99**	0.8	**0.97**
Radius M–L displacement	1.5	**0.86**	0.6	**0.91**	0.9	**0.91**

**Table 3:** Ligament peak load throughout the simulation period for different velocity conditions.

Ligament	Bundles	Peak Ligament Load (N)		
		10 deg/s	60 deg/s	Free Velocity
MCL anterior part	Anterior	23	37	101
	Central	45	61	123
	Posterior	61	76	112
MCL posterior part	Anterior	35	40	55
	Central	30	44	87
	Posterior	24	47	125
LUCL	Anterior	43	58	80
	Central	23	33	44
	Posterior	26	25	50
RCL	Anterior	21	44	131
	Central	18	26	55
	Posterior	12	31	88
Annular ligament	Proximal	46	51	52
	Distal	36	41	41

## References

[1] Morrey B.F. (2000). The Elbow and Its Disorders.

[2] Garner B.A., Pandy M.G. (2001). Musculoskeletal model of the upper limb based on the visible human male dataset. Comput. Methods Biomech. Biomed. Eng..

[3] Gonzalez R.V., Hutchins E.L., Barr R.E., Abraham L.D. (1996). Development and evaluation of a musculoskeletal model of the elbow joint complex. J. Biomech. Eng..

[4] Holzbaur K.R., Murray W.M., Delp S.L. (2005). A model of the upper extremity for simulating musculoskeletal surgery and analyzing neuromuscular control. Ann. Biomed. Eng..

[5] Willing R.T., Nishiwaki M., Johnson J.A., King G.J., Athwal G.S. (2014). Evaluation of a computational model to predict elbow range of motion. Comput. Aided Surg..

[6] Benham M.P., Wright D.K., Bibb R. (2001). Modelling soft tissue for kinematic analysis of multi-segment human body models. Biomed. Sci. Instrum..

[7] Tanaka S., An K.-N., Morrey B.F. (1998). Kinematics and laxity of ulnohumeral joint under valgus-varus stress. J. Musculoskelet. Res..

[8] Fisk J.P., Wayne J.S. (2009). Development and validation of a computational musculoskeletal model of the elbow and forearm. Ann. Biomed. Eng..

[9] Asraf Ali M., Sundaraj K., Badlishah Ahmad R., Ahamed N.U., Islam A. (2014). Recent observations in surface electromyography recording of triceps brachii muscle in patients and athletes. Appl. Bionics Biomech..

[10] Van Woensel W., Arwert H. (1993). Effects of external load and abduction angle on emg level of shoulder muscles during isometric action. Electromyogr. Clin. Neurophysiol..

[11] Cignoni P., Callieri M., Corsini M., Dellepiane M., Ganovelli F., Ranzuglia G. Meshlab: An open-source mesh processing tool. Proceedings of the Sixth Eurographics Italian Chapter Conference.

[12] Donahue T.L., Hull M.L., Rashid M.M., Jacobs C.R. (2002). A finite element model of the human knee joint for the study of tibio-femoral contact. J. Biomech. Eng..

[13] Zielinska B., Donahue T.L. (2006). 3D finite element model of meniscectomy: Changes in joint contact behavior. J. Biomech. Eng..

[14] Rahman M., Cil A., Johnson M., Lu Y., Guess T.M. (2014). Development and validation of a computational multibody model of the elbow joint. Adv. Biomech. Appl..

[15] Rahman M., Cil A., Bogener J.W., Stylianou A.P. (2016). Lateral collateral ligament deficiency of the elbow joint: A modeling approach. J. Orthop. Res..

[16] Miyake J., Moritomo H., Masatomi T., Kataoka T., Murase T., Yoshikawa H., Sugamoto K. (2012). In vivo and 3-dimensional functional anatomy of the anterior bundle of the medial collateral ligament of the elbow. J. Shoulder Elb. Surg..

[17] Morrey B.F., An K.N. (1985). Functional anatomy of the ligaments of the elbow. Clin. Orthop. Relat. Res..

[18] Blankevoort L., Huiskes R. (1991). Ligament-bone interaction in a three-dimensional model of the knee. J. Biomech. Eng..

[19] Wismans J., Veldpaus F., Janssen J., Huson A., Struben P. (1980). A three-dimensional mathematical model of the knee-joint. J. Biomech..

[20] Li G., Gil J., Kanamori A., Woo S.L. (1999). A validated three-dimensional computational model of a human knee joint. J. Biomech. Eng..

[21] Blankevoort L., Kuiper J.H., Huiskes R., Grootenboer H.J. (1991). Articular contact in a three-dimensional model of the knee. J. Biomech..

[22] Regan W.D., Korinek S.L., Morrey B.F., An K.N. (1991). Biomechanical study of ligaments around the elbow joint. Clin. Orthop. Relat. Res..

[23] Bloemker K.H., Guess T.M., Maletsky L., Dodd K. (2012). Computational knee ligament modeling using experimentally determined zero-load lengths. Open Biomed. Eng. J..

[24] Rahman M., Cil A., Stylianou A.P. (2016). Prediction of elbow joint contact mechanics in the multibody framework. Med. Eng. Phys..

[25] Guess T.M., Stylianou A.P., Kia M. (2014). Concurrent prediction of muscle and tibiofemoral contact forces during treadmill gait. J. Biomech. Eng..

[26] Kia M., Stylianou A.P., Guess T.M. (2014). Evaluation of a musculoskeletal model with prosthetic knee through six experimental gait trials. Med. Eng. Phys..

[27] Renani M.S., Rahman M., Cil A., Stylianou A.P. (2018). Calibrating multibody ulno-humeral joint cartilage using a validated finite element model. Multibody Syst. Dyn..

[28] Stylianou A.P., Guess T.M., Cook J.L. (2014). Development and validation of a multi-body model of the canine stifle joint. Comput. Methods Biomech. Biomed. Eng..

[29] Stylianou A.P., Guess T.M., Kia M. (2013). Multibody muscle driven model of an instrumented prosthetic knee during squat and toe rise motions. J. Biomech. Eng..

[30] Renani M.S., Rahman M., Cil A., Stylianou A.P. (2017). Ulna-humerus contact mechanics: Finite element analysis and experimental measurements using a tactile pressure sensor. Med. Eng. Phys..

[31] Carmichael M.G., Liu D. (2015). Upper limb strength estimation of physically impaired persons using a musculoskeletal model: A sensitivity analysis. Conf. Proc. IEEE Eng. Med. Biol. Soc..

[32] Gonzalez R.V., Abraham L.D., Barr R.E., Buchanan T.S. (1999). Muscle activity in rapid multi-degree-of-freedom elbow movements: Solutions from a musculoskeletal model. Biol. Cybern..

[33] Gonzalez R.V., Andritsos M.J., Barr R.E., Abraham L.D. (1993). Comparison of experimental and predicted muscle activation patterns in ballistic elbow joint movements. Biomed. Sci. Instrum..

[34] O’Driscoll S.W., An K.N., Korinek S., Morrey B.F. (1992). Kinematics of semi-constrained total elbow arthroplasty. J. Bone Jt. Surg. Br..

[35] Morrey B.F., An K.N. (1983). Articular and ligamentous contributions to the stability of the elbow joint. Am. J. Sports Med..

[36] Pigeon P., Yahia L., Feldman A.G. (1996). Moment arms and lengths of human upper limb muscles as functions of joint angles. J. Biomech..

[37] Eckstein F., Merz B., Muller-Gerbl M., Holzknecht N., Pleier M., Putz R. (1995). Morphomechanics of the humero-ulnar joint: II. Concave incongruity determines the distribution of load and subchondral mineralization. Anat. Rec..

[38] Brand R.A. (2005). Joint contact stress: A reasonable surrogate for biological processes?. Iowa Orthop. J..

